# Breaking down the tumor immune infiltration within pediatric sarcomas

**DOI:** 10.3389/fendo.2023.1187289

**Published:** 2023-06-22

**Authors:** Rachel Weil, David Loeb

**Affiliations:** ^1^ Department of Developmental and Molecular Biology, Albert Einstein College of Medicine, Bronx, NY, United States; ^2^ Department of Pediatrics, Albert Einstein College of Medicine, Bronx, NY, United States

**Keywords:** osteosarcoma, Ewing sarcoma, immunotherapy, macrophage, T cell

## Abstract

Immunotherapies are a promising therapeutic option, yet for a variety of reasons, these treatments have achieved limited success against sarcomas. The immunosuppressive tumor microenvironment (TME) of sarcomas as well as lack of predictive biomarkers, decreased T-cell clonal frequency, and high expression of immunosuppressive infiltrating cells has thus far prevented major success using immunotherapies. By breaking down the TME into its individual components and understanding how the various cell types interact with each other as well as in the context of the complex immune microenvironment, can lead to effective therapeutic immunotherapy treatments, potentially improving outcomes for those with metastatic disease.

## Introduction

### Pediatric bone sarcomas

Sarcomas are among the most common solid tumors in children and adolescents, accounting for 13% of cancers in patients under 20 (1). Osteosarcoma (OS) and Ewing sarcoma (EWS) are the most common bone tumors in children and adolescents. Osteosarcoma is the most common malignant bone tumor commonly diagnosed in children and young adults with a median age of 16. OS is derived from bone-forming mesenchymal cells and is characterized by TP53 and RB inactivation as well as massive chromosomal rearrangements derived through the process of chromothripsis. Tumors commonly present in the metaphysis of long bones, specifically in the distal femur or proximal tibia, with the most common site of metastasis being the lungs. For patients with localized disease, 5-year event free survival (EFS) is around 65%, whereas patients who present with metastatic disease or who relapse have worse 5-year EFS, at about 25% and less than 20% respectively. Current standard of care treatment comprises surgical resection of detectable disease in conjunction with systemic chemotherapy (2–5). Ewing sarcoma is the second most common type of primary bone cancer, composed of small round blue cells thought to be of either neural crest or mesenchymal lineage (6). EWS is molecularly defined by chromosomal translocations between a *FET* gene family member fused with an *ETS* transcription factor, with the most common fusion being Ewing sarcoma breakpoint region 1 protein-Friend leukemia integration 1 (*EWSR1-FLI1*). Tumors occur predominantly in the pelvis, femur, tibia and ribs. Like osteosarcoma, patients with localized disease have a 5-year overall survival rate between 65-75%, yet those presenting with metastatic disease have a 5-year overall survival rate of less than 30% and those with recurrence have a 5-year overall survival rate of approximately 10% (7). Standard of care treatment typically starts with chemotherapy with primary tumor control by surgery and/or radiation (1, 2, 8).

Despite attempts to treat metastatic disease more aggressively, outcomes have not improved for several decades, emphasizing the need for improved understanding of the biology behind these tumors as well as identifying novel therapeutic targets to develop treatments that more effectively treat patients with metastatic disease. Immunotherapies are a promising therapeutic option, yet for a variety of reasons, these treatments have achieved limited success against sarcomas. Sarcomas have an immunosuppressive tumor microenvironment (TME), so gaining a more comprehensive picture of the tumor immune microenvironment is critical for developing ways to improve the efficacy of immunotherapies. Although bone sarcomas have relatively low tumor mutational burden, especially compared to most carcinomas, because pediatric immune systems are more robust and cellular compared to adults, immunotherapy remains a very promising avenue for improving survival in this population. For both adults and children with sarcomas, lack of predictive biomarkers, decreased T-cell clonal frequency, and high expression of immunosuppressive infiltrating cells has thus far prevented major success using immunotherapies. Through a more detailed understanding of the TME in conjunction with identification of novel immune targets, immunotherapy holds promise as a beneficial therapeutic, potentially improving outcomes for those with metastatic disease.

## Sarcoma TME

The bone microenvironment is highly dynamic, composed of bone cells (osteoclasts, osteoblasts, osteocytes), stromal cells (mesenchymal stromal cells, fibroblast), vascular cells (endothelial cells, pericytes), immunes cells (macrophages, lymphocytes) and a mineralized extracellular matrix (ECM) (9). Under physiological conditions, these various cell populations coordinate to maintain healthy bone homeostasis through paracrine and autocrine communications. Tumor cells can manipulate and disrupt this complex ecosystem and transform the environment to one that promotes their survival and growth. The tumor microenvironment consists of mesenchymal cells, tumor infiltrating cells, endothelial cells, extracellular matrix molecules, and inflammatory mediators (10). Immune cells play a key role in mediating the crosstalk between sarcomas and the bone microenvironment, contributing to controlling both tumor growth and cell extravasation and metastasis. Tumor infiltrating cells of both the adaptive and innate immune systems such as tumor associated macrophages (TAMs), T lymphocytes, dendritic cells, B lymphocytes, and mast cells, make up the tumor immune microenvironment of sarcomas (11). Sarcoma cells influence the recruitment and differentiation of immune infiltrating cells, establishing an immunosuppressive environment that promotes tumor growth and metastases.

## Innate immunity

**Figure 1:**
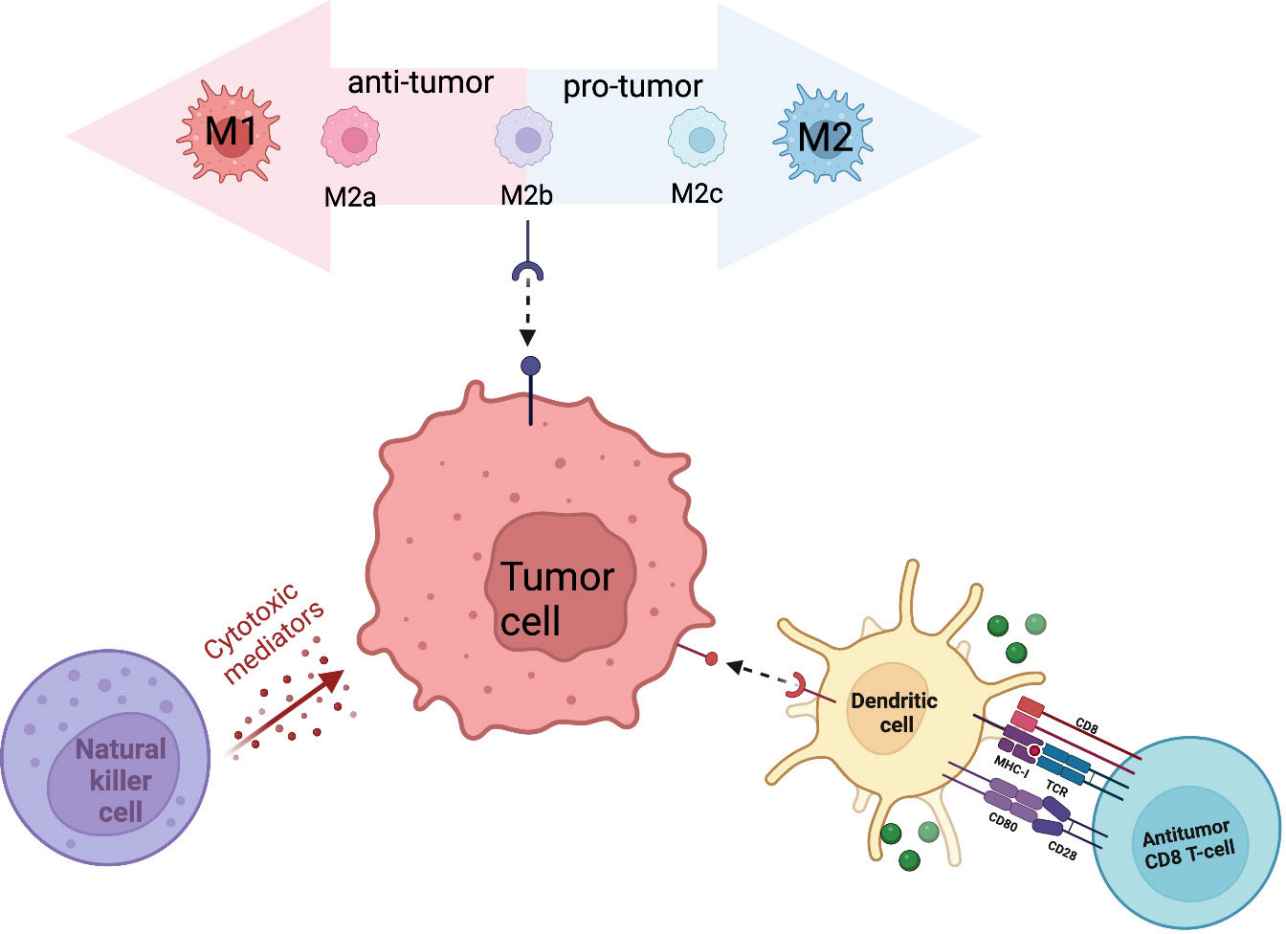
Primary innate immune cells (macrophages, NK cells, DCs) that contribute to the sarcoma tumor immune microenvironment.

Innate immune cells serve as the first line of defense in anti-tumor immunity. Through direct recognition and killing, innate immune cells contribute to tumor suppression while simultaneously triggering an adaptive immune response. The sarcoma immune microenvironment contains multiple innate immune cell types including dendritic cells, macrophages, and natural killer cells. The antitumor properties of innate immune cells, along with their increased abundance in sarcomas, designates them as a target for cell-based therapy (**Figure 1**).

### Macrophages

In the context of normal bone biology, highly specialized macrophages, osteoclasts, are involved in bone resorption and contribute to maintaining bone homeostasis (12). In the context of the tumor microenvironment, macrophages, referred to as tumor associated macrophages (TAMs), function in mediating angiogenesis, regulating tumor cell migration and invasion, and metastatic spread. TAMs are made up of a large subpopulation of M1 and M2 type macrophages that span a polarization spectrum in response to specific stimuli and environmental cues. M1-polarized macrophages are anti-tumor cells whereas M2-polarized macrophages act as pro-tumor regulators (13). While M1 and M2 serve as the most extreme polarized states of macrophages, macrophages pass through multiple iterations of M2 type macrophages. M2a and M2b macrophages play an immunomodulatory role and promote T-helper 2 cell response. Upon transition to M2c, these macrophages are associated with immune response suppression and tissue remodeling (14). A dysregulation between M1 and M2-TAMs in favor of M1 macrophages was detected in non-metastatic patients (12), whereas an increased M2-phenotype was associated with worse prognosis (15). Multiple studies have observed that M2-polarized TAMs contribute to tumor growth and metastatic spread by suppressing intra-tumor T-lymphocytes. Depletion of M2-TAMs led to an increase of T-lymphocytes proliferation and associated increase of pro-inflammatory cytokines (16). Aggressive pediatric bone sarcomas exhibited an increased number of peripheral CD14+HLA-DRlow/neg immunosuppressive monocytes as well as an increase of cytotoxic T-Lymphocyte Associated protein 4 (CTLA4+) and CD14+ macrophage infiltrates (17). The number of M2-TAMs was correlated with the frequency of suppressive T-cell immunoglobulin and mucin-domain containing-3 (TIM-3+) programmed cell death 1 (PD-1)+ T lymphocytes in osteosarcoma patients (14, 18). Conversely, several studies have found TAMs to be associated with metastatic inhibition and that high levels of M2-polarized macrophages are related to longer metastasis progression free survival (16, 19, 20). The exact role TAMs play in propelling metastatic disease has been challenging to discern in part due to the dynamic polarization of infiltrating macrophages, eclipsing full representation of their role in the TME. Signaling pathways play a key role in TAM reprogramming so better elucidating the significance of different pathways in macrophage repolarization can aid in developing therapies that regulate polarization and conversion from M2 to M1-like TAMs (14). High-resolution technologies such as multiplex immunohistochemistry (mIHC), cytometry by time of flight (CyTOF), high-throughput scRNA-seq, and spatial transcriptomics can aid in investigating the distinct TAM subtypes and capture the distinct stages of polarization rather than focusing only on the extremes of M1 and M2 (21). Integrating various applications provides complementary profiles on phenotype, transcriptome, and infiltration status.

While there is contradictory evidence as to the exact role of TAMs in the TME, it is clear they play a prominent role in driving tumor growth and are an attractive therapeutic target. Suppressing the M2 phenotype or preventing M2 polarization through pharmacological therapy has been shown to be beneficial in treating osteosarcomas (22–24). An alternate avenue to utilize macrophages to suppress tumor growth is through recruitment of non-TAM macrophages. Upregulation of Secreted Protein, Acidic and Rich in Cysteine-like 1 (SPARCL1) protein induces osteosarcoma cells to secrete chemokine ligand 5 (CCL5), leading to macrophage infiltration. These recruited macrophages were shown to inhibit osteosarcoma metastasis (25). Activating the tumoricidal properties of macrophages to inhibit cell growth has shown to improve overall survival. Mifamurtide, a macrophage activator composed of phosphatidyl choline and phosphatidyl serine liposomes containing muramyl tripeptide phosphatidyl ethanolamine (L-MTP-PE), is an immunostimulant that activates monocytes and macrophages to exert antitumor effects. The incorporation of mifamurtide has resulted in improvement in 6- and 5- year overall survivals, when added to chemotherapy in nonmetastatic and metastatic osteosarcoma patients, respectively (26–28). Although not FDA-approved, mifamurtide is approved for use in the European Union. A recently completed clinical trial (NCT01525602) tested the efficacy of pexidartinib, a small molecule inhibitor of CSF-1R, which has been implicated in recruiting monocytes to the TME leading to their differentiation into TAMs. Chemotherapy triggers an increased expression in CSF-1R, recruiting TAMs and contributing to chemotherapy resistance. The combination of pexidartinib and paclitaxel was well tolerated and found to block CSF-1R signaling, indicating a reliable strategy to diminish macrophage tumor infiltration (29). Currently, patients are being recruited to a Phase 2 clinical trial (NCT02502786) focused on what effect combination GM-CSF and humanized antibody 3F8 has on preventing recurrence. 3F8 is an antibody that attaches to the GD2 protein on cancer cells and helps focus immune cells to attack cancer cells. GM-CSF is a protein that increases the number of white blood cells that can be targeted to kill cancer cells and is thought to enhance the effectiveness of 3F8.

### Natural killer cells

Natural Killer (NK) cells express both activating and inhibitory receptors that are capable of recognizing target cells without prior sensitization. Once bound to the target cell, the cytolytic activity of NK cells is activated to remove tumor cells. NK cells are also able to recruit dendritic cells into the TME, further contributing to tumor lysis (30, 31). Inhibitory surface receptors include killer-cell immunoglobulin receptors (KIR), which recognize specific human leucocyte antigen (HLA) class I molecules HLA-A, B, and C, as well as CD94/NK group 2 member A (NKG2A), which recognizes HLA-E. NK cell activating receptors include natural cytotoxic receptors (NCRs) and NK group 2 member D (NKG2D) that recognize stress proteins like Major histocompatibility complex class I chain-related protein A/B (MICA/B) and UL16-binding protein (ULBP) (32). NK cell activity promotes an anti-tumor response through the release of cytotoxic granules containing granzymes and perforin, expression of death receptor ligands on their surface, and the recruitment and production of cytokines. Typically, patients diagnosed with sarcomas have low levels of NK cells. NK cell numbers and activity can be increased through combination of surgery and polychemotherapy with IL-2 administration. The stimulated increase correlates with positive clinical outcomes, indicating the anti-tumor activity and key function in immune surveillance of NK cells (33). Activation is particularly successful in osteosarcoma patients, as osteosarcoma cells have high expression of CD54 and CD58 cell-surface molecules, allowing for easy detection and stronger binding with NK cells (34–36). Adoptive cell therapy is the primary therapeutic approach that has been taken to the development of NK cell therapy. This involves isolating cells from patients, manipulating them ex vivo, and then reinfusing them back into the patient. To maximize effectiveness, adoptive NK cell transfer is commonly accompanied with additive treatment options. Epigenetic drugs like histone deacetylase inhibitors and DNA-methylation inhibitors increase the expression of ligands for activating receptors on NK cells or death receptors on the sarcoma cells augmenting NK cells cytotoxic capabilities (37–40). Multiple clinical trials using epigenetic drugs focused on targeting DNA methylation (NCT04195555, NCT03600649, NCT05266196, NCT03514407, NCT02712905) and histone deacetylase inhibitors (NCT04025931). Standard chemotherapy drugs have been shown to increase NK cell-activating ligand expression in tumors (41) (42),. Cytokine therapy can aid in enhancing binding between NK cells and tumor cells through upregulating expression of CD18, CD2, intracellular adhesion molecule (ICAM)-1, and fibronectin (43–45). IL-15 and IL-2 have been shown to enhance the cytolytic activity of NK cells (20, 46–50). Monoclonal antibodies that target specific NK cell receptors can enhance overall cytotoxicity. NK cell targeted cell lysis occurs through antibody-dependent cell-mediated cytotoxicity (ADCC) mediated by their CD16 (FcyRIIIa) receptors. Targeting receptors known to be upregulated on tumor cells with an antibody that contains the Fc region that bind to CD16 on NK cells amplifies ADCC enhancing NK mediated cell lysis (49). CAR-NK cells, while difficult to engineer, provide the added benefit of being able to detect tumor-associated antigen levels too low to trigger ADCC (51). While no clinic trials using CAR-NK cells for sarcoma patients exist, pre-clinical data and clinical trials using CAR-NK cells in other cancer types demonstrate enhanced cytotoxicity (52, 53). Though not yet recruiting, a currently active Phase I trial (NCT02890758) is looking to find the number of natural killer cells from non-HLA matched donors that can be safely infused into patients with cancer. NK cells are being donated by healthy individuals and are not HLA matched. Patients receiving NK cells may also be given a drug called ALT803 to help keep NK cells alive and aid their cancer fighting characteristics. A Phase 2 trial (NCT02100891) is using HLA-haploidentical hematopoietic cell transplantation (HCT) followed by an early, post-transplant infusion of donor NK cells on day +7 with the hope of providing a mechanism to treat high-risk solid tumors, leading to improved disease control rate. The belief is that the infusion of donor NK cells will influence the development of NK and T cell subtypes which will provide immediate/long-term tumor surveillance, infectious monitoring, and durable engraftment.

### Dendritic cells

DCs act as a bridge between innate and adaptive immunity by functioning as antigen presenting cells (APCs), taking up cell-associated antigens to present them to CD8+ T cells. Dendritic cells also function to activate other innate immune cells such as cytokine induced killer cells (54). Dendritic cells develop from immature to mature cells, with distinct functions and phenotypes at each stage. Immature DCs possess phagocytic activity and reside in peripheral tissue where they capture and process antigens. Upon exposure to pathogen-derived products or innate pro-inflammatory signals, DCs migrate to draining lymph nodes where they lose endocytic activity and become mature DCs. Upon maturation, DCs interact with antigen-specific T cells triggering a targeted immune response. Disturbance of DC differentiation, survival, and function is commonly seen in tumors, with fewer DCs being detected in Ewing sarcomas compared to osteosarcomas, likely due to the overall reduced TAM and T-lymphocyte populations (9). Suppression or depletion of this cell type contributes to immune tolerance to tumor antigens in sarcomas, specifically due to alterations of carbohydrates on the cell surface interfering with the interaction between C-type lectins on DCs and tumor cells, interfering with the overall antigen presentation process (55, 56). To combat the immunosuppression caused by the deprivation of antigens presented, DC vaccines have been developed to compensate for the lack of APCs. DCs isolated from peripheral mononuclear cells are matured and loaded with ex vivo tumor antigens and then infused back into the patient, where the antigen activated DCs should boost the immune response. When tested in rats and mice, DC vaccines showed success in inhibiting primary and metastatic tumor growth, while also decreasing the amount of regulatory T lymphocytes, reducing levels of immunosuppressive cytokines, and increasing the CD8+ T lymphocyte population (57, 58). Unfortunately, when brought to clinical trial, while well tolerated, DC vaccines did not yield effective results (59). Prior treatments, such as chemotherapy, can damage innate and adaptive immune effectors, limiting their availability and efficacy to respond to the increased antigen presentation. Lack of migration of effector cells to the tumor site due to down-regulation of chemokine expression as well as other strong immunosuppressive mechanisms likely contribute to the lack of success of DC vaccines. Current clinical trials are attempting to combat these issues by combining DC vaccines with other treatments to yield maximum efficacy. A currently active phase 1 trial of autologous dendritic cell vaccination with imiquimod immunomodulation in children and adults with refractory sarcoma (NCT01803152) is showing this approach to be feasible and well tolerated (60). The DC vaccine is administered intradermally in imiquimod-treated skin, which serves as a vaccine adjuvant to propel DC activation and increase systemic immune responses to the vaccine (61). Further assessment is needed to evaluate initial and sustained antitumor activity following vaccine administration.

## Adaptive immunity

T-lymphocytes, particularly CD8+ T lymphocytes and CD20+ B lymphocytes, comprise much of the infiltrate within osteosarcomas. Tumor infiltrating lymphocytes (TILs) are detectable in 75% of osteosarcomas with a peak around 86% in metastases (62). Several factors contribute to the ineffective immune response to sarcomas despite the infiltration of lymphocytes, including a lack of tumor neo-antigens, low expression of immunomodulatory molecules, and secretion of suppressive mediators that inhibit activation and expansion of TILs. Much research has focused on immune checkpoints that serve as modulators of anti-tumor T cell response. A major modulator of T-lymphocytes are ligands of the B7 protein family. Osteosarcoma cells express several subtypes from the B7 family including HHLA2 and PDL-1 proteins (63, 64). HHLA2 expression was detected at higher levels in metastatic specimens and correlated with metastatic disease and poor survival, suggesting that HHLA2 contributes to immunosuppression. Sarcoma cells expressing B7-H3 correlate with aggressive and metastatic disease (56). PDL-1 and PDL-2 expressed on osteosarcoma cells bind to PD-1 expressed on TILs, contributing to immunosuppression and tumor progression (65–68). In Ewing sarcoma, MHC class I molecule HLA-G is expressed on both tumor cells and tumor-infiltrating lymphocytes (69). HLA-G is associated with an increased number of TILs and its upregulation can contribute to immune escape (70).

Immune checkpoints are responsible for moderating the activation and function of immune populations and inhibit anti-tumor immunity. Blockade of PD-1/PD-L1 leads to the expansion of exhausted intratumoral CD8+ T cells. In osteosarcoma, PD-L1 expression positively correlates with infiltration of NK cells, T cells, and dendritic cells, as well as poor 5-year event free survival (71). However, for immune checkpoint inhibitors to be effective, there needs to be a significant presence of the checkpoint molecule and OS and EWS express little PD-L1 on tumor cells (72). Despite lack of PD-L1 expression, immune checkpoint blockade has potential to be effective when given in combination with PD-L1 expression stimulators (73). Clinical trials using checkpoint inhibitors that target PD-L1 have shown little response in patients with osteosarcoma and no response in those with Ewing sarcoma. While response rates were low with combination of CTLA-4 and PD-1 blockade in metastatic sarcoma patients, they showed significantly improved response rates compared to those receiving PD-1 monotherapy (74). Interestingly, two types of adult sarcomas, undifferentiated pleomorphic sarcoma and dedifferentiated liposarcoma, exhibited strong enough response to lead to expanded cohorts (75). The discrepancy in response reaffirms the differences in treating adult versus pediatric disease, largely due to the increased neoantigen burden in adults (71). Even with the multiple therapies aimed at targeting PDL-1, success remains poor, leading to the development of therapies targeting different checkpoint molecules. HHLA2 (B7-H7), a B7 family member, is involved in regulating T cell function (76). As a co-inhibitory molecule, B7-H7 expression in osteosarcomas is associated with poor survival and metastatic disease (77). A current clinical trial (NCT02982941) is studying the efficacy of enoblituzumab in children with B7-H3 expressing tumors, including Ewing sarcoma and osteosarcoma.

**Figure 2:**
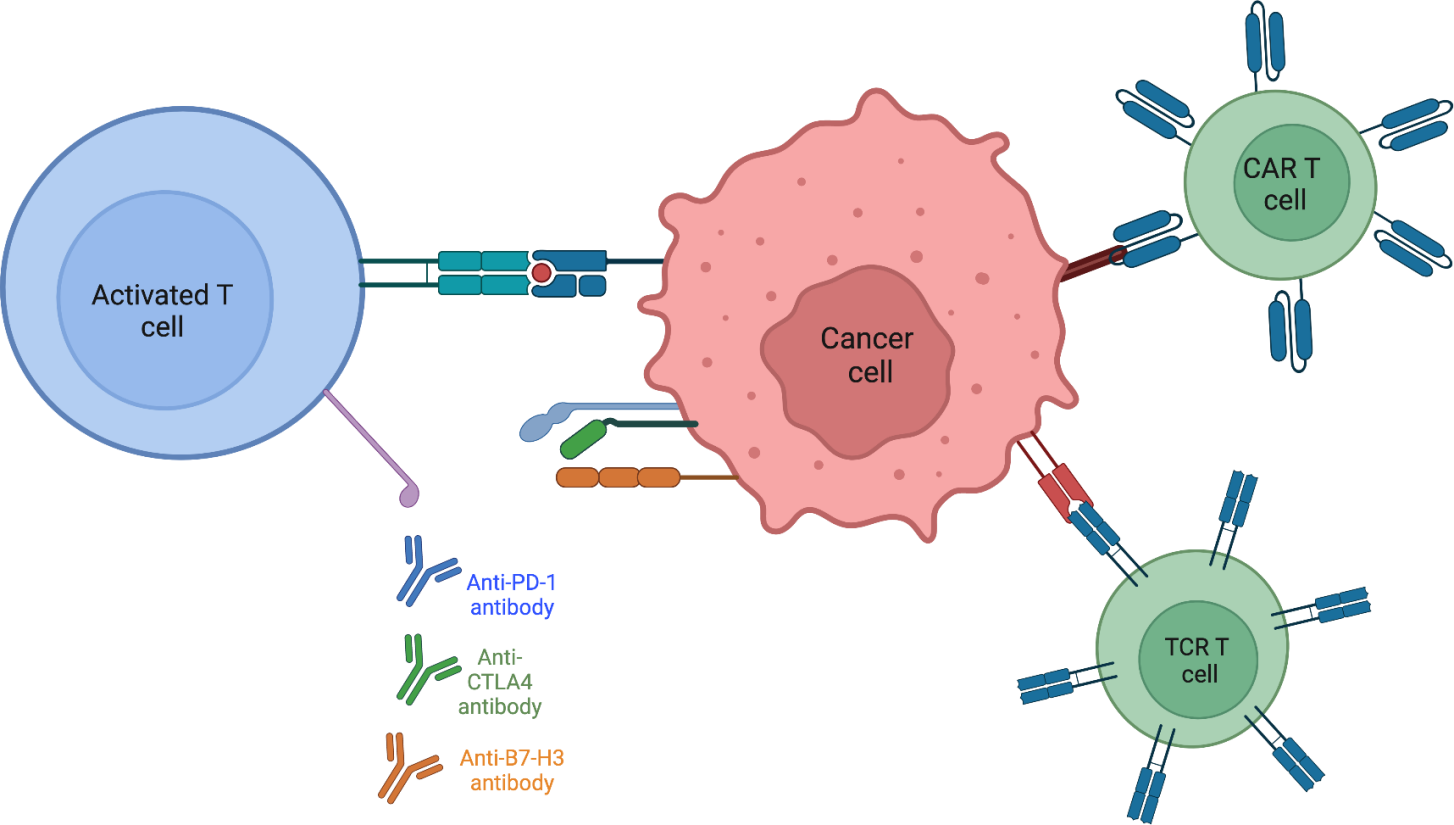
Activating present T-cells within the tumor immune microenvironment is aided by targeting various checkpoints known to be expressed on sarcoma tumor cells. Engineering T-cells to directly recognize and destroy tumor cells or by modifying the T-cell receptor itself to circumvent surface antigen expression by binding to MHC.

Autologous T-cells with enhanced activity against specific tumor epitopes is a way to alter the tumor microenvironment (78). Rather than receptor antibody mediated modification, as in the case of anti-PD1 therapy, a T-cell targeted approach allows for the direct introduction of known tumor antigens that ideally activate the immune system against the tumor. Chimeric antibody receptor engineered T cell (CAR-T) therapy trains immune cells to recognize, locate, and kill tumor cells. While this method has shown success in leukemia and lymphoma, solid tumors present problems involving penetration of CAR-T cells into the tumor (79, 80). Antigen heterogeneity within solid tumors makes it more difficult for T cells to detect cancer cells and mutes the effectiveness of CAR-T therapy. Both the range of expression as well as varying levels of antigen expression make it challenging to design CAR-T cells which are engineered to target cell specific tumor associated antigens. Whereas CAR-T cells return to the bloodstream and have ample contact with blood tumor cells, in solid tumors CAR-T cells are not easily able to infiltrate tumor tissue. The combination of solid tumor composed of dense matrix, secreting less vascular-related factors, and reduction in chemokines that could aid CAR-T cell movement, causes penetration to remain poor. The often-immunosuppressive microenvironment of solid tumors creates a hostile environment, restricting the function of CAR-T cells (81). CAR-T cell clinical trials for sarcomas are focusing design on classes of targets unique to pediatric sarcomas (82). Receptor tyrosine kinases are an attractive CAR-T target as many are overexpressed. Specifically, CAR-T cells directed against the human epidermal growth factor receptor 2 (HER2) are being developed, as HER2 is expressed in many sarcoma subtypes. One study (NCT00902044) found no evident toxicities with 3 out of 16 patients with recurrent or refractory osteosarcoma achieving stable disease for 12 to 15 weeks and following tumor resection, remained in remission. Epidermal growth factor (EGFR) is another RTK that may serve as a promising CAR-T target. A phase I clinical trial (NCT03618381) is currently recruiting children with EGFR positive tumors. This trial also combines the EGFR specific CAR with a CD19-specific CAR with the hypothesis that CD19 B cells will act as an antigen presenting cell to T cells, promoting their expansion and survival. After showing tumor regression in preclinical mouse xenograft models of osteosarcoma and Ewing sarcoma (83), a phase I clinical trial (NCT04483778) using B7-H3 CAR-T cells is currently recruiting patients with B7-H3 expressing tumors. To circumvent issues from CAR-T cell therapies, T-cell receptor (TCR) therapies utilize the natural mechanism of T cells to recognize cancer cells, providing more potential as an effective therapy in sarcomas. Whereas CAR-T cells can only recognize extracellular targets, TCRs are designed to recognize tumor-specific epitopes presented by the major histocompatibility complex (MHC) molecules on the tumor cell surface. This broadens the range of TCR targets as they are not limited to tumor-specific proteins on the cell surface, but can recognize tumor-specific sequences within a cell presented by the MHC (84). The ability of TCRs to target intracellular proteins (such as fusion oncoproteins) offers an improved/expanded opportunity to treat solid tumors (**Figure 2**).

## Combination immunotherapy

Due to the complexity of the tumor immune microenvironment, only targeting one cell population is not enough, rather some form of combination therapy that activates multiple cascades to work in tandem to induce an effective and cytotoxic immune response is likely to be more successful. Multiple immune checkpoint molecules are expressed on innate immune cells and targeting these in combination can influence both innate and adaptive immune cells, reversing exhaustion and inhibiting tumor growth and spread. From studies on glioblastoma patients, it was found that those who had tumor infiltrating lymphocytes with a higher PD-1+/CD8+ ratio had worse response to DC vaccines (85). The PD-1/PD-L1 axis was exhausting DC vaccine-primed CD8+ T cells, contributing to the low success of DC vaccines in osteosarcoma patients, indicating that PD-1 inhibitors might improve the efficacy of DC vaccines (86). An ex vivo study found that anti-PD-1 treatment enhanced T-cell responses induced by DC vaccines fused with myeloma cells (87). Melanoma bearing mice exhibited increased anti-tumor activity when treated with anti-PD-1 treatment as it increased the function and infiltration of TILs induced by vaccines (86). The PD-1/PD-L1 axis inhibits NK cell response but combining PD-1 blockade with NK cell transfusion can more effectively target tumor cells (88, 89). PD-1+ TAMs exhibit an M2-like profile and function and are able to suppress T cell function (90, 91). In a LM7 osteosarcoma mouse model, macrophages in lung metastases highly express PD-1, and PD-1 blockade decreased the number of lung nodules by increasing the macrophage tumor infiltration and polarization from M2 to M1 (92). While less studied, combining CTLA-4 blockade with innate immune cell regulators has the potential to mount a more potent response. Current clinical trials testing the effect of a synthetic mRNA-electroporated DC vaccine with ipilimumab (anti-CTLA-4 antibody) has shown promising response in patients with advanced melanoma (93). CTLA-4 has been detected on tumor infiltrating NK cells in mice but has yet to be looked at in humans so further studies are needed to assess the benefit of combined CTLA-4 blockade and NK cell-based therapy (94). Innate and adaptive immune cells express TIM-3, which is also co-expressed with PD-1 (95). Cells bearing TIM-3-PD-1 are significantly more exhausted than those with PD-1 alone and correlate with poor prognosis (96) (97). TIM-3 expression on NK cells and macrophages can contribute to their exhaustive and inhibitory state, raising the possibility that targeting TIM-3 could also be a valuable therapeutic approach, augmenting NK cell function.

## Remaining questions

Despite attempts to characterize and identify components of the tumor immune microenvironment, response to immunotherapies remains low in most sarcoma patients. Many questions remain as to why exactly the response is so low, primarily surrounding immune resistance. Identifying mechanisms of resistance is crucial to yielding success when using immunotherapies. Breaking down the components of what may be contributing to immune resistance can aid in better guiding research to focus on specific avenues that can lead to meaningful, applicable findings. By nature, the sarcoma TME is immunosuppressive. Better understanding the delicate balance of infiltrating immune cells can guide treatment to transform the TME to one of an immunocompetent nature. Due to the heterogeneity of sarcomas, mapping out the various cascades of tumor infiltration and suppression within different sarcoma settings (particularly considering age) is key to developing strategies that utilize the right combination of therapies that exploit immune cells to effectively kill tumor cells. As sarcomas are stromal/mesenchymal tumors, defining the mechanism by which the stroma itself can drive resistance can provide important context to understanding not only which immune cells are able to not only infiltrate the tumor, but also how they act. Tumor mutational burden (TMB) contributes to immunotherapy response through generation of immunogenic neo-peptides expressed on MHC on the tumor cell surface. Sarcomas are characterized by a low tumor mutational burden which is thought to contribute to low immunogenicity. However, the underlying mechanism between the association between TMB and immunotherapy success has yet to be elucidated. TMB has been shown to be a predictive biomarker, specifically for immune checkpoint inhibitors (98). Improved diagnostic methods and refining TMB’s role in eliciting an immune response can contribute to developing a more targeted immunotherapy approach. Biomarkers, while not fully explaining mechanisms of resistance can aid in guiding treatment plans. Detecting cells of interest can further characterize immune response within different sarcoma settings and not only contribute to specific therapy development but better characterize responses amongst different tumor settings.

## Future directions

Although much remains to be discovered concerning the makeup and function of the tumor immune microenvironment, what is clear is the need for combination therapies to yield successful therapeutic effects. With improvements in genomic analysis and advanced sequencing, more thorough research can be done into elucidating underlying immune resistance within sarcomas. Immunogenomic profiling aids in identifying immune-associated sarcoma subtypes and thus better identify patients who would benefit from immunotherapy. Transcriptome based studies are useful in characterizing distinct molecular features such as immune signaling cascades and other key genes that contribute to and validate an immune-associated prognostic biosignature. Focusing therapies on not just the immune cells present, but on stimulating an immune response to manipulate the TME broadens the availability and potential for success of future treatment options. While heterogeneity within sarcomas makes them challenging to treat because of the variability and complexity of interacting cells, it also provides an opportunity to expand treatment from what we believe are targetable cells, providing more opportunity for successful clinical outcomes.

## Author contributions

RW wrote the first draft, and she and DL jointly edited. All authors contributed to the article and approved the submitted version.
